# Case Report: Cutaneous  metastases indicative of endometrial cancer: A case report and review of the literature

**DOI:** 10.12688/f1000research.75527.1

**Published:** 2022-02-16

**Authors:** Olfa Zoukar, Anis Haddad, Yosra Jemaa, Ines Zouari, Rahma Issa, Amel Bayar, Ghada Khouildi, Amal Sebri, Dalel Naguez, Asma Boukadida, Mossaab Ghannouchi, Raja Faleh

**Affiliations:** 1 Department of Gynecology and Obstetrics, University Hospital Fattouma Bourguiba, Monastir, 5000, Tunisia; 2 Department of Gynecology and Obstetrics, Hospital HAJ ALI SOUA Ksar Hellel, ksar hlel, monastir, Tunisia; 3 Department of Gynecology and Obstetrics, University, Hospital Taher SFAR Mahdia, mahdia, Tunisia; 4 Department of General Surgery, University Hospital Taher SFAR Mahdia, mahdia, Tunisia

**Keywords:** cutaneous metastases, endometrial cancer, poor prognosis, case report

## Abstract

Secondary localizations of cutaneous metastases from endometrial cancer are rarely observed with a prevalence of 0.8% and can be indicative of deep pelvic cancer (ovarian or endometrial). The prognosis is usually poor, with skin metastases most often indicating advanced disease.

This work, based on a case observed in our department and a review of the literature, aims to highlight the existence of this dramatic form of cutaneous extension of a common disease. Dermatologists are often consulted due to the non-specific nature of the lesions and should be aware of this entity. As with other cutaneous metastases, an accurate diagnosis is based on a the patient’s thorough medical and surgical history in conjunction with histopathology

## Introduction

Endometrial cancer is the third pelvic gynecological malignancy in Tunisia after breast and cervical cancer. It occurs most commonly in postmenopausal with 60 years of age being the frequency peak. However, up to 25% of cases can appear before menopause. ^1^

Its evolution is long and locoregional. Exceptionally, distant metastases to the abdominal wall, the lungs and bones can be revealing.

Cutaneous metastases are rarely observed with a prevalence of 0.8%. ^2^ They most often present with an umbilical lesion, Sister Marie Joseph nodule. The spread of metastasis most often occurs via the lymphatic route, therefore close to the organ of origin, but it can also occur through the hematogenous route, therefore affecting distant organs or contiguity cutaneous metastases are typically painless when they are neither large nor infected. They have well-defined edges and are usually hard and attached to the subcutaneous tissues, covered with normal skin or slightly hyperemic (increased vascularization in the tumor) as long as the lesion does not become ulcerated. In the presence of cutaneous metastases, the prognosis is usually poor, with skin metastases most often indicating advanced disease. Almost half of these patients die within six months of the discovery of cutaneous metastases, the darkest prognosis accompanying cutaneous metastases from lung cancer.

Hereby, We report the case of a 61-year-old woman with an umbilical swelling which appeared six months prior to presentation secondarily ulcerated and oozing. She also reported an unintentional weight loss and a deterioration of her overall health with repetitive metrorrhagia. Biopsy of this lesion revealed the characteristics.

A literature review is also provided.

## Case report

A 61-year-old patient, house wife, with personal medical history of dyslipidemia and hypertension, obese with BMI 31, with no relevant family medical history, consulted her dermatologist for a budding peri-umbilical skin lesion that appeared six months before, initially neglected by the patient.

**Figure 1.**
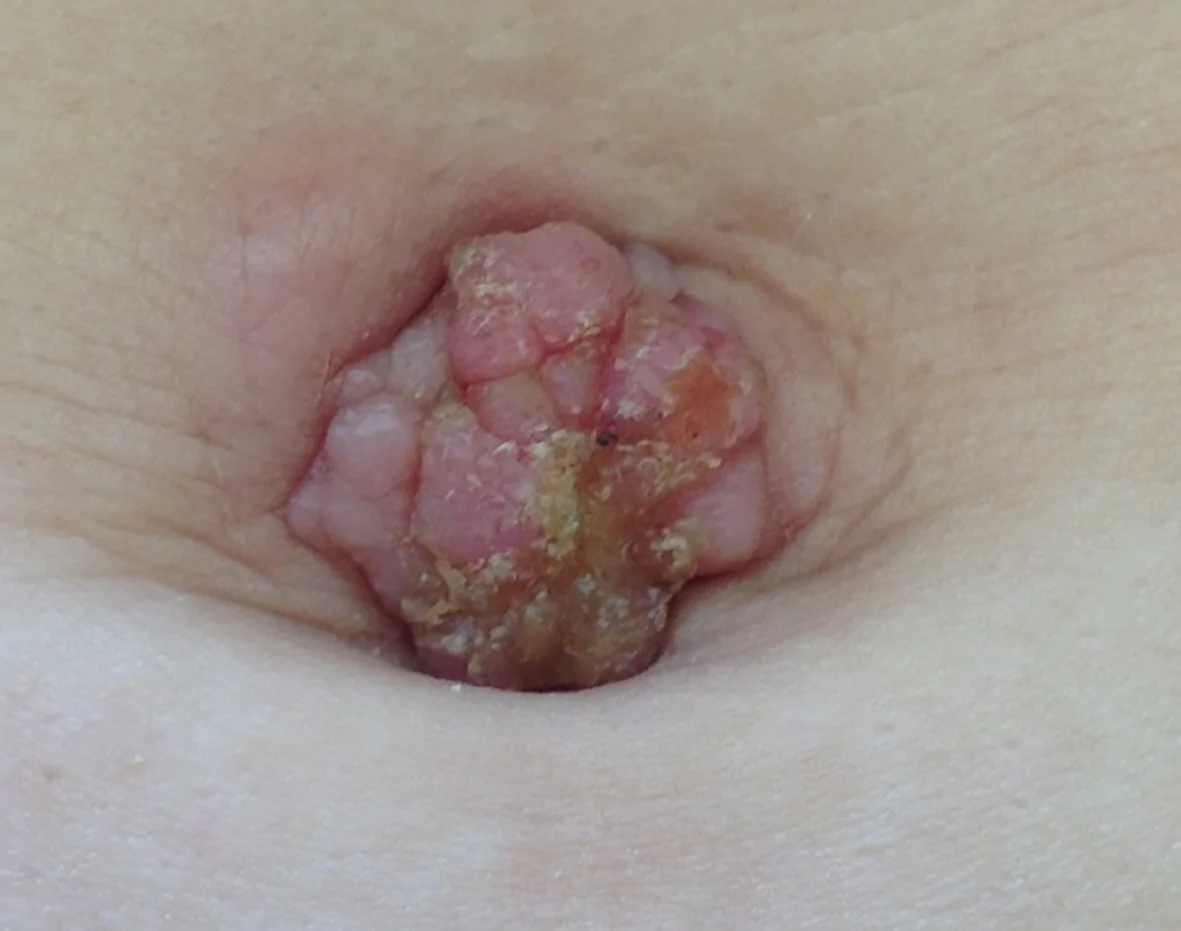
Sister Marie Joseph's nodule localization of endometrial cancer.

This clinical picture was associated with diffuse abdominopelvic pain with no colonic transit disorder. The patient reported unexplored repeated metrorrhagia. Physical examination showed a deterioration of the patient’s overall condition to 3 according to the WHO performance index, a painful, ulcerated and oozing umbilical nodule measuring 3 cm in diameter along its longest axis ( Figure 1).

In addition, there was free ascites of low abundance and tumor-like hepatomegaly. The abdominal ultrasound revealed hetero-multi-nodular hepatomegaly of secondary appearance and ascites of low abundance. The umbilical skin lesion was biopsied and the pathology study concluded that there was a cutaneous metastasis of an adenocarcinoma whose histological appearance was compatible with a well differentiated endometrioid endometrial adenocarcinoma.

The patient was referred our department for additional treatment. On examination, the patient was apyretic, pale, asthenic, complaining of exertional dyspnea (walking perimeter of a few meters) and presented with a budding nodular skin lesion measuring 3 cm on the major axis of foul odor and bleeding in the biopsy area. The pelvic examinations revealed indurated rectovaginal septum with an enlarged uterus.

Biological tests showed severe hypochromic microcytic anemia due to iron deficiency at 3.5 g/dl and tumor markers, in particular ACE, were increased. Pelvic ultrasound revealed a uterus of normal morphology but increased in size with a very thickened endometrium. The appendices were not seen with the presence of a low abundance effusion in the pouch of Douglas. The thoroco-abdominopelvic CT confirmed the ultrasound data with the presence of secondary lesions in the lungs and liver.

A biopsy and hemostatic curettage of the endometrium was carried out after transfusion of 4 globular pellets and the anatomopathological study substantiated the presence of well differentiated endometrioid endometrial adenocarcinoma.

An abdominopelvic MRI performed as part of the extension assessment highlighted the presence of multiple pelvic iliac and lumboaortic lymphadenopathies with infiltration of nearby organs and secondary liver and digestive lesions.

The endometrial tumor was classified as T4B N2 M1 and the patient was non-operable and received 4 cycles of chemotherapy with pelvic external radiotherapy of 50 Gy. The evolution was unfavorable and death occurred 6 months later.

## Discussion

Cutaneous metastases occur in 0.6% to 10.4% of all cancer patients. The frequency of Cutaneous metastases is correlated with the frequency of each malignant tumor, which is why women with skin metastases most often have the following primary malignant tumors: breast, ovary cavity, lung and large intestine. Globally, skin metastases represent 2% of all skin neoplasms. ^3^^–^^5^

These lesions may be the only manifestation of an underlying visceral cancer.

A systematic search of the English-language literature on PubMed between 1966 and 2013 identified only 26 cases of skin metastases in endometrial cancer. ^6^^,^^7^ Most of these reports highlighted the rarity of this dissemination model.

Skin metastasis of endometrial cancer is associated with a poor prognosis and an average life expectancy of approximately 4 to 12 months after diagnosis. ^8^^,^^9^ It can take any form of lesion, including nodules, papules, ulcers and plaques. In our case, biopsy allowed us to confirm the presence of skin metastases related to endometrial cancer.

## Conclusion

Our patient represents a dramatic form of skin extension of a common disease. Dermatologists are often consulted due to the non-specific nature of the lesions and should be aware of this entity. As with other skin metastases, a thorough medical and surgical history in conjunction with histopathology is necessary for an accurate diagnosis.

## Data availability

All data underlying the results are available as part of the article and no additional source data are required.

## Author contributions

All the authors contributed to the conduct of this work. All authors also declare that they have read and approved the final version of the manuscript.

## Consent

Written informed consent for publication of their clinical details and/or clinical images was obtained from the patient before death and further consent was obtained from his husband after his death.
