# Supplementary material for: Case Report: Cutaneous  metastases indicative of endometrial cancer: A case report and review of the literature
Source: F1000Res. 2022 Feb 16;11:194. [Version 1] doi: 10.12688/f1000research.75527.1 (PMC13554742; doi:10.12688/f1000research.75527.1)
Supplement: Peer review history

# Reviewer report: Reviewer response for version 1

**DOI:** 10.5256/f1000research.79410.r503951

**Published:** 2026-07-28

**Authors:** Phuc Nhon Nguyen

**Affiliations:** 1 Tu Du Hospital, Ho Chi Minh City, Vietnam

The first paragraph should be mention endometrial cancer in the general context instead of author's country. 

 This sentence lacks of reference citation. "Its evolution is long and locoregional. Exceptionally, distant metastases to the abdominal wall, the lungs and bones can be revealing."

 Please mention role of Doppler ultrasound in assessing endometrial cancer. 

 [Reference 4 ]doi: 10.5468/ogs.22053.

 [Reference 5] doi: 10.1007/s40477-022-00732-w. 

 MRI should be added to investigate completely. 

 Uterine sarcomas should be excluded by imaging modalities. [Reference 3] doi: 10.1002/jcu.24046. 

 Histology should be added to presentation case.

 Discussion should be expanded with mechanism and risk factors.

 Similar cases to cutaneous metastases of endometrial cancer should be summarized in a table.

 Some previous papers have mentioned on this rare entity. [Reference 2] doi: https://doi.org/10.4021/jcgo6e

 Management should be discussed. Please read this paper.[Reference 1] https://doi.org/10.3390/diagnostics13152603

 All references should be presented in English database.

Are enough details provided of any physical examination and diagnostic tests, treatment given and outcomes?

Partly

Is the case presented with sufficient detail to be useful for other practitioners?

Partly

Is sufficient discussion included of the importance of the findings and their relevance to future understanding of disease processes, diagnosis or treatment?

Partly

Is the background of the case’s history and progression described in sufficient detail?

Partly

Reviewer Expertise:

Gynecologist

I confirm that I have read this submission and believe that I have an appropriate level of expertise to confirm that it is of an acceptable scientific standard, however I have significant reservations, as outlined above.

## References

[1] : Cutaneous Metastasis of Endometrial Cancer and Long-Term Survival: A Scoping Review and Our Experience. *Diagnostics* .2023;13(15) : 10.3390/diagnostics13152603 10.3390/diagnostics13152603 PMC1041762637568966

[2] : Cutaneous Metastasis of Endometrial Carcinoma: A Case Report and Literature Review. *Journal of Clinical Gynecology and Obstetrics* .2012; 10.4021/jcgo6e 10.4021/jcgo6e

[3] : Assessing the Clinical Characteristics and the Role of Imaging Modalities in Uterine Sarcoma: A Single‐Center Retrospective Study From Vietnam. *Journal of Clinical Ultrasound* .2025;53(7) : 10.1002/jcu.24046 1527-1537 10.1002/jcu.24046 40312923

[4] : Endometrial thickness and uterine artery Doppler parameters as soft markers for prediction of endometrial cancer in postmenopausal bleeding women: a cross-sectional study at tertiary referral hospitals from Vietnam. *Obstetrics & Gynecology Science* .2022;65(5) : 10.5468/ogs.22053 430-440 10.5468/ogs.22053 PMC948367035488358

[5] : Additional value of Doppler ultrasound to B-mode ultrasound in assessing for uterine intracavitary pathologies among perimenopausal and postmenopausal bleeding women: a multicentre prospective observational study in Vietnam. *Journal of Ultrasound* .2022;26(2) : 10.1007/s40477-022-00732-w 459-469 10.1007/s40477-022-00732-w PMC1024793236284050

# Reviewer report: Reviewer response for version 1

**DOI:** 10.5256/f1000research.79410.r188624

**Published:** 2023-08-17

**Authors:** Ottavia D’Oria

**Affiliations:** 1 Department of Medical and Surgical Sciences and Translational Medicine, Sapienza University, Sapienza, Italy

*It has been identified that this reviewer report does not meet F1000's requirements as outlined in the editorial policies [https://f1000research.com/about/policies]. The authors have been advised not to use this review report when revising this article, but it will remain a permanent part of the article audit trail.*

 I read with great interest the manuscript, which falls within the aim of this Journal and offers a high-quality overview of the topic. Although the manuscript can be considered already of high quality, I would suggest taking into account the following minor recommendations:

- I suggest another round of language revision, in order to correct a few typos and improve readability.
- The introduction should be extended and completed. I find it interesting to include a reference to the molecular classification of endometrial cancer and to its implications to personalize the therapeutic path of patients with EC (see PMID: **36979434 and **PMID: **36833105**).
- I also suggest authors to better organize the discussion section following this ideal structure: main findings of the study, strength and limitations of the study, implications and comparison with literature, and future directions.

Are enough details provided of any physical examination and diagnostic tests, treatment given and outcomes?

Yes

Is the case presented with sufficient detail to be useful for other practitioners?

Partly

Is sufficient discussion included of the importance of the findings and their relevance to future understanding of disease processes, diagnosis or treatment?

Partly

Is the background of the case’s history and progression described in sufficient detail?

Yes

Reviewer Expertise:

Gynecologic Oncology

I confirm that I have read this submission and believe that I have an appropriate level of expertise to confirm that it is of an acceptable scientific standard, however I have significant reservations, as outlined above.

## References

[1] : Novel Insights into Molecular Mechanisms of Endometrial Diseases. *Biomolecules* .2023;13(3) : 10.3390/biom13030499 10.3390/biom13030499 36979434 PMC10046407

[2] : Role of Genomic and Molecular Biology in the Modulation of the Treatment of Endometrial Cancer: Narrative Review and Perspectives. *Healthcare (Basel)* .2023;11(4) : 10.3390/healthcare11040571 10.3390/healthcare11040571 36833105 PMC9957190

# Reviewer report: Reviewer response for version 1

**DOI:** 10.5256/f1000research.79410.r124048

**Published:** 2022-03-07

**Authors:** Fethi Derbel

**Affiliations:** 1 Department of Surgery, University of Sousse, Sousse, Tunisia

This article shows the delay in diagnosing an endometrial adenocarcinoma. The author describes the case report very well. The diagnosis delay was very well emphasised by the authors, however, the authors should give some recommendations for gynecologists and patients to make the diagnosis of endometrial adenocarcinoma earlier, before there is metastasis.

 The message in this article was mainly given to dermatologists to be aware of cutaneous metastasis.

 The review of the literature concerning this article is up-to-date and the article is appropriate to be indexed without modifications.

Are enough details provided of any physical examination and diagnostic tests, treatment given and outcomes?

Yes

Is the case presented with sufficient detail to be useful for other practitioners?

Yes

Is sufficient discussion included of the importance of the findings and their relevance to future understanding of disease processes, diagnosis or treatment?

Yes

Is the background of the case’s history and progression described in sufficient detail?

Yes

Reviewer Expertise:

I am a professor of Surgery, my area of research is mainly cancers

I confirm that I have read this submission and believe that I have an appropriate level of expertise to confirm that it is of an acceptable scientific standard.
